# A Prospective, Multicenter, Single-Group Target-Value Clinical Trial to Evaluate the Safety and Efficacy of a Large Bore Aspiration Catheter System for the Endovascular Treatment of Acute Ischemic Stroke

**DOI:** 10.3389/fneur.2022.864563

**Published:** 2022-06-09

**Authors:** Fei Chen, Xuesong Bai, Wei Hu, Fuqiang Guo, Jian Chen, Zhiming Zhou, Yanfei Chen, Peng Gao, Yongchang Liu, Qingwu Yang, Xinfeng Liu, Yun Xu, Huisheng Chen, Yabing Wang, Bin Yang, Adam A. Dmytriw, Aman B. Patel, Qingfeng Ma, Liqun Jiao

**Affiliations:** ^1^Department of Neurology, Xuanwu Hospital, Capital Medical University, Beijing, China; ^2^Department of Neurosurgery, Xuanwu Hospital, Capital Medical University, Beijing, China; ^3^China International Neuroscience Institute (China-INI), Beijing, China; ^4^Department of Neurology, Anhui Provincial Hospital, Anhui Medical University Hefei, Anhui, China; ^5^Department of Neurology, Sichuan Provincial People's Hospital, Chengdu, China; ^6^Department of Neurology, The First Affiliated Hospital of Wannan Medical College, Wuhu, China; ^7^Department of Neurovascular Intervention, Cangzhou Central Hospital, Cangzhou, China; ^8^Department of Neurology, Xinqiao Hospital and The Second Affiliated Hospital, Army Medical University (Third Military Medical University), Chongqing, China; ^9^Department of Neurology, Eastern Theater General Hospital of the Chinese People's Liberation Army, Nanjing, China; ^10^Department of Neurology, Drum Tower Hospital, Medical School of Nanjing University, Nanjing, China; ^11^Department of Neurology, General Hospital of Northern Theater Command, Shenyang, China; ^12^Neuroendovascular Program, Massachusetts General Hospital, Harvard Medical School, Beijing, China; ^13^Department of Interventional Neuroradiology, Xuanwu Hospital, Capital Medical University, Beijing, China

**Keywords:** acute ischemic stroke, large vessel occlusion, mechanical thrombectomy, aspiration, efficacy

## Abstract

**Objective:**

This study was conducted to determine the safety and efficacy of the Esperance^®^ Distal Access Catheter (0.071”), a novel large bore aspiration catheter in treating acute ischemic stroke (AIS) with large vessel occlusion (LVO).

**Methods:**

A prospective multicenter clinical trial involving 15 stroke centers was performed. Baseline characteristics, procedural data, and angiographic and clinical outcomes of all acute stroke procedures (from May 2020 to March 2021) using the novel large bore aspiration catheter were analyzed.

**Results:**

During the study period, 160 consecutive patients were recruited. The mean age and median baseline NIHSS were 65.4 years and 16, respectively. Successful reperfusion was achieved in 147 (91.9%) cases [at least modified Thrombolysis in Cerebral Infarction (mTICI) 2b] with aspiration alone, with complete reperfusion (mTICI 3) in 94 (58.8%) cases. Successful/complete first pass reperfusion was achieved in 104 (64.60%) cases, including mTICI 2b in 34 (21.1%) cases and mTICI 3 in 70 (43.5%) cases. The time from groin puncture to successful reperfusion was 44 (33, 62) min. There were 16 (9.9%) cases requiring rescue therapy using stent-retriever. Procedure-related complications included 2 (1.3%) cases of arterial perforation, 2 (1.3%) cases of arterial dissection, 12 (7.5%) cases of distal embolization, and 1(0.6%) case of puncture site infection. The rate of symptomatic intracranial hemorrhage (sICH) was 3.8% and mortality rate was 13.8%. A total of 99 (62.3%) cases had a favorable outcome (mRS 0–2) at 90 days.

**Conclusions:**

In current practice, the first-line aspiration approach with the Esperance^®^ Distal Access Catheter is safe and efficacious. This device may achieve high reperfusion rates with lower instances of rescue stent retriever therapy.

## Introduction

Acute ischemic stroke (AIS) has been considered one of the major causes of mortality and morbidity worldwide. In 2015, a series of randomized controlled trials (RCTs) proved the superiority of mechanical thrombectomy (MT) over standard medical therapy in treating patients with acute ischemic stroke (AIS) and large vessel occlusion (LVO) in the anterior circulation (1). Later trials further extended the efficacy of MT in treating AIS patients with extended time-window (2, 3). As such, recent guidelines from the American Heart Association/American Stroke Association (AHA/ASA) recommended MT as the first-line therapy for AIS-LVO (4).

The continued breakthrough of MT largely relies on the advancement of mechanical devices. Thrombectomy devices, with distinct mechanisms of action, may directly impact clinical consequences. MT was performed using stent retriever (SR) of the second-generation in previous RCTs (1). Thereafter, a direct aspiration first pass technique (ADAPT), with a large-bore catheter to aspirate the thrombus, was shown to have comparable effectiveness when compared with first-line SR in the Contact Aspiration vs. Stent Retriever for successful Revascularization (ASTER) trial (5) and the Comparison of Direct Aspiration Vs. Stent Retriever as a First Approach (COMPASS) (6) trial. Thus, direct aspiration thrombectomy as first-pass MT is currently considered as noninferior to SR for patients with AIS-LVO patients (COR I, LOE B-R) by AHA/ASA guidelines (4).

Aspiration force and efficacy was reported to be closely related with catheter inner diameter (ID) (7). Thus, technical advancements in catheters mainly focused on improving the deliverability as well as larger ID at both the distal end that engages the thrombus as well as the proximal end. Currently, several large-bore aspiration catheters are available on the market with refinements, in large part driven by technological advancements, such as Sofia Plus 0.070”, (MicroVention, California, USA), ACE64 (0.064”), and ACE68 (0.068”) catheters (Penumbra, California, USA) (7, 8). To better meet the rapidly growing demand for thrombectomy devices in China, several aspiration devices have been approved by the China Food and Drug Administration (CFDA), but few of them have been industrially manufactured in the country. The aim of this study was to investigate the safety and effectiveness of the Esperance^®^ Distal Access Catheter 0.071” (Wallaby, Shanghai, CN), a novel large bore aspiration catheter in treating AIS based on multicenter data.

## Methods

### Study Design

This study was a prospective multicenter clinical trial (Chinese Clinical Trial Registry, ChiCTR2000031463) and was funded by Shanghai Wallaby Medical Technology Co., Ltd. Patients who received mechanical thrombectomy using the Esperance^®^ Distal Access Catheter 0.071” in 15 participating centers were recruited. Approval of the local ethics committee of the participating centers was obtained. All patients or their legal custodians were informed about the nature, purpose, and potential risks of the trial and signed informed consent forms before participation. This clinical trial was conducted under the Good Clinical Practice regulations (GCP). The trial passed the Shanghai FDA inspection and quality check of each participating site according to the clinical trial quality check of each hospital.

### Inclusion Criteria

Subjects were eligible when they meet all of the following criteria: Age ≥ 18; acute ischemic onset stroke, preoperative imaging (CTA/DSA/MRA) confirmed anterior circulation occlusion (internal carotid artery or middle cerebral artery M1 segment or M2 segment occlusion, M2 segment occlusions were pursued according to physician judgement) or posterior circulation (vertebrobasilar or P1) vascular occlusion; NIHSS score ≥6;mRS ≤2 before stroke onset; ASPECTS (acute anterior circulation stroke) or pc-ASPECTS (acute posterior circulation stroke) ≥ 6 points; within 24 h from stroke onset; informed consent form signed by subjects or their legal guardian. Exclusion criteria for patients were: anterior circulation large area cerebral infarction (infarct volume ≥ 70 ml or infarct area> 1/3 MCA blood supply area) or focal space occupying lesion effect significantly led to displacement of the midline structure; intracranial hemorrhage or subarachnoid hemorrhage; simultaneous acute occlusion of bilateral carotid arteries; tandem lesions with simultaneous occlusion of intracranial and extracranial vessels; occlusion of an intracranial artery due to arterial dissection or arteritis; subjects who are participating in clinical studies of other drugs or devices. Further details of the inclusion and exclusion criteria are shown in Supplementary Material.

### Intervention

The indication for treatment was left to the discretion of each operator according to local protocol. Bridging intravenous thrombolysis before thrombectomy was administered in patients treated within 4.5 h of stroke onset in the absence of contraindication. MT was performed under local anesthesia alone, conscious sedation, or general anesthesia depending on patient condition and each contributing center anesthesia protocol. For all selected cases, the operator decision was to perform distal aspiration through the Esperance^®^ Distal Access Catheter (0.071”) as the first pass. The choice of the Esperance^®^ Distal Access Catheter (0.071”) relies on several aspects including operator discretion, local protocols, catheter availability, and imaging characteristics fitting with the ADAPT strategy as a first-line treatment strategy.

First, an 8F guide catheter (Cordis Corporation, Miami, FL) was placed into the cervical internal carotid artery or vertebral artery. Then, an Esperance^®^ Distal Access 6F catheter was advanced to the occlusion site with or without microwire and microcatheter support, depending on tortuosity of vessels. A more than 1-min aspiration period, using either a dedicated pump (Medela AG) or manual depression through a vacuum syringe, was applied before catheter removal. In the case of failure or partial efficacy, this maneuver could be repeated twice as desired. In case of ADAPT failure or insufficient recanalization, the choice to convert to another endovascular technique (distal aspiration combined with stent retrievers or stent retrievers alone) was left to operator discretion. Complementary endovascular treatments, such as cervical, vertebral, or intracranial angioplasty and/or stent placement, were performed depending on stroke etiology and severity as well as angiographically detected underlying lesions.

### Data Collection

Both clinical and radiologic data were collected. Baseline data included age, sex, comorbidities (e.g., hypertension [HTN], diabetes mellitus [DM], dyslipidemia, atrial fibrillation [AF]), history of smoking, history of drinking, baseline National Institutes of Health Stroke Scale (NIHSS), administration of intravenous thrombolysis, etc. Imaging data included baseline ASPECTS from non-contrast computed tomography (NCCT) and anterior circulation occlusion site diagnosed from initial CTA and confirmed with digital subtraction angiography (DSA). All imaging, including DSA, pre- and post-treatment CT, and post-treatment MRI (as available), were separately analyzed by a central Core Lab.

### Trial Outcomes

The primary efficacy endpoint was successful reperfusion (defined as modified Thrombolysis in Cerebral Infarction [mTICI modified] 2b-3) of target vessels after aspiration only.

The secondary efficacy endpoints were complete reperfusion (mTICI 3) of target vessels after the first aspiration (first pass effect, FPE); successful reperfusion (mTICI 2b-3) of target vessels after the first aspiration (modified first pass effect, mFPE); time from arterial puncture to successful reperfusion of target vessels; the proportion of rescue therapy using SR; final successful reperfusion rate (allowing the use of adjunctive therapy); change in NIHSS 24–36 h after intervention; change in NIHSS 7 days after intervention or at discharge (whichever occurs first); favorable clinical outcome (modified Rankin Scale 0 to 2) at 90 days and the success rate of catheter use.

The safety endpoints were mortality (all cause and stroke-related) within 90 days; the recurrence of stroke within 90 days; postoperative (24–36 h) symptomatic intracranial hemorrhage (sICH) and any type of intracranial hemorrhage (ICH) and surgical complications (distal embolism, arterial dissection, arterial perforation, etc.).

### Statistical Analysis

Categorical variables are expressed by frequency and percentages. Continuous variables were expressed by their means and standard deviations, or by the median and the interquartile range (IQR) in the case of non-normal distribution. The normality of distributions was assessed graphically and the Shapiro-Wilk test was used. All statistical analyses were performed using SAS 9.4 (SAS Institute, Cary, NC, USA).

## Results

### Baseline Characteristics of Patients

From May 2020 to March 2021, a total of 160 consecutive patients, including 91 males and 69 females, were treated for large vessel occlusion using the Esperance^®^ Distal Access Catheter 0.071”. Baseline characteristics are shown in Table 1. The mean age of the patients was 65.4 ± 11.9 years. The details of demographic and vascular risk factors are shown in Table 1. The baseline NIHSS of the recruited patients were 16 (13, 20). A total of 52 (32.5%) patients received bridging thrombolysis. Regarding occlusion site, the majority of procedures were performed in the anterior circulation, including 43 (26.9%) cases of ICA, 87 (54.4%) cases of M1, 9 (5.6%) cases of M2, and 1 (0.63%) of ACA occlusion. There were 19 (11.9%) cases of vertebrobasilar and 2 (1.3%) of PCA occlusion. The time from symptom onset to first diagnostic angiogram was 342.8 (241.5, 355.0) min.

**Table 1:** Demographic and baseline characteristics.

**Demographics**	**N = 160**
Age (years), mean (SD)	65.4(11.9)
Sex, male	91 (56.9%)
Past medical history	
HTN	98 (61.3%)
DM	31 (19.4%)
Dyslipidemia	40 (25.0%)
CAD	34 (21.3%)
AF	67 (41.9%)
Smoking	
Current smoker	39 (24.4%)
Previous smoker	11 (6.9%)
Past stroke history	39 (24.4%)
PAD	1 (0.6%)
Clinical data	
IV-tPA	52 (32.5%)
Baseline NIHSS, median (Q25, Q75)	16 (13, 20)
Time from symptom onset to artery puncture (min), median (Q25, Q75)	342.8 (241.5, 355.0)
Occluded location	
ICA	43 (26.9%)
M1	87 (54.4%)
M2	9 (5.6%)
Vertebrobasilar	19 (11.9%)
PCA	2 (1.3%)

*HTN, hypertension; DM, diabetes mellitus; AF, atrial fibrillation; CAD, coronary artery disease; PAD, peripheral artery disease; NIHSS, National Institutes of Health Stroke Scale; IV-tPA, intravenous tissue plasminogen activator; ICA, internal carotid artery; PCA, posterior cerebral artery; SD, standard deviation*.

### Outcomes

Details of the outcomes are shown in Table 2. Regarding primary efficacy, 147 (91.9%) cases had successful reperfusion with aspiration alone, with mTICI 2b in 53 (33.1%) cases and mTICI 3 in 94 (58.8%) cases. Regarding the secondary efficacy, 99 (62.3%) cases had favorable outcome (mRS 0–2) at 90 days. Successful/complete reperfusion with FPE was achieved in 104 (64.60%) cases, including mTICI 2b in 34 (21.1%) cases and mTICI 3 in 70 (43.5%) cases. The time from groin puncture to successful reperfusion was 44 (33,62) min. There were 16 (9.9%) cases requiring stent-retriever rescue therapy. A total of 158 (98.1%) cases achieved successful reperfusion. When compared with baseline NIHSS, the decrease of NIHSS was 4 (9) and 9 (14), respectively at 24–36 h after surgery and 7 days or at discharge. Overall, 159 (99.4%) cases had technically successful aspiration catheter operation.

**Table 2:** Primary and secondary outcomes.

**Outcomes**	
Primary efficacy	
Angiographic outcome after sole aspiration, mTICI	
0	8 (5.0%)
1	0 (0.0%)
2a	5 (3.1%)
2b	53 (33.1%)
3	94 (58.8%)
Successful reperfusion with sole aspiration	147 (91.9%)
Secondary efficacy	
Favorable outcome (mRS 0–2) at 90 days	99 (62.3%)
Angiographic outcome first pass, mTICI	
0	30 (18.6%)
1	14 (8.7%)
2a	13 (8.1%)
2b	34 (21.1%)
3	70 (43.5%)
Complete reperfusion after first pass (FPE)	70 (43.5%)
Successful reperfusion after first pass (mFPE)	104 (64.6%)
Puncture to successful reperfusion time (min), median (Q25, Q75)	44 (33,62)
Rescue therapy using SR	16 (9.9%)
Final successful reperfusion	158 (98.1%)
NIHSS, median (Q25, Q75)	
24–36 h	11 (6,18)
7 days or at discharge	9 (3,17)
ΔNIHSS compared to baseline, median (IQR)	
24–36 h	4 (9)
7 days or at discharge	9 (14)
Successful use of device	159 (99.4%)
Safety	
Any ICH	30 (18.9%)
Symptomatic ICH	6 (3.8%)
Mortality at 90 days	
All cause	22 (13.8%)
Stroke related	13 (8.1%)
Recurrent stroke	0 (0.0%)
Procedure-related complications	
Perforation	2 (1.3%)
Dissection	2 (1.3%)
Distal embolization	12 (7.5%)
Puncture site infection	1 (0.6%)
Others	1 (0.6%)

*mTICI, modified Thrombolysis in Cerebral Infarction; mRS, modified Rankin Scale; NIHSS, National Institutes of Health Stroke Scale; IQR, interquartile rang; ICH, intracranial hemorrhage*.

Regarding safety endpoints, the rate of any ICH within 24–36 h after intervention was 18.9% (30 cases), and sICH was 3.8% (6 cases). The 90-day mortality rate was 13.8%. Procedure-related complications were 2 (1.3%) cases of arterial perforation, 2 (1.3%) cases of arterial dissection, 12 (7.5%) cases of distal embolization, and 1 (0.6%) case of puncture site infection.

## Discussion

Results from this clinical trial support the safety and efficacy of the Esperance^®^ Distal Access Catheter (0.071”) in AIS patients with LVO, treated with the ADAPT technique. Of the 160 patients, 147(91.9%) cases had successful reperfusion (mTICI 2b-3) with aspiration alone, and 99 (62.3%) cases had a favorable outcome (mRS 0–2) at 90 days. The time between groin puncture to successful revascularization was 44 (IQR 33,62) min. Regarding safety endpoints, the morbidity rate at 90 days was favorable 13.8% and the sICH rate within 24–36 h after procedure was 3.8%. The results were similar to previous reports from the PROMISE study using ACE68/ACE64 catheters (9).

In the past 5 years, ADAPT has become commonly employed as a first-line choice for treating AIS-LVO. The Esperance^®^ Distal Access Catheter, a novel large bore aspiration catheter, has a larger internal diameter (0.071”), which enable both greater aspiration force and faster aspiration speed to address the challenges of direct aspiration in the context of high thrombus load. This aspiration device also demonstrates excellent flexibility and trackability, owing to its thinner wall (0.005”) and smaller outer diameter (0.0810”), to traverse tortuous vessels and access vascular lesions during intervention. This new catheter system allows for rapid and effective revascularization by using a variety of endovascular techniques, with excellent kink resistance.

Larger bore catheters may have several advantages, such as convenience of use, higher rates of achieving successful reperfusion, lower rates of rescue therapy, and faster time to revascularization (10). In a retrospective database among 510 patients with the largest experience of ADAPT, catheters with a larger diameter could yield more frequent technical success and better clinical outcomes when compared with smaller catheters (7). Successful reperfusion with aspiration alone was 85% using ACE 64.064” and with 81% ACE 68.068”, and the use of SR was respectively 29 and 28%. In a recent meta-analysis of first-line Sofia catheters, the successful reperfusion rate was 71.6% (95%CI, 66.3–76.5%) with an SR rescue rate of 24.1% (95%CI, 17.7–31.9%). The overall final successful reperfusion rate was 88.9% (95%CI, 82.6–93.1%) (11). At the same time, in previous studies using SR, relatively lower successful reperfusion rates were reported [HERMES meta-analysis 71%, (1) ASTER 83.1%, (5) COMPASS 89% (6)]. The recently published ASTER-2 trial showed successful reperfusion was 86.2% in the combined approach group and 72.3% in the SR alone group (12). In our study, the rate of successful reperfusion with aspiration alone and at final angiography was 91.9 and 98.14%, with a lower rescue rate using SR (9.9%). Thus, the Wallaby aspiration catheter system was shown with better, or at the very least, comparable efficacy in successful revascularization of occluded vessels compared with other modern thrombectomy devices.

The FPE and mFPE are new metrics to evaluate the efficacy of thrombectomy devices (13). Previous meta-analyses reported better clinical outcomes with FPE or mFPE when compared with a multiple-pass effect (MPE) in patients with AIS-LVO (14). The mFPE was achieved in 69.7% of cases in a multicenter retrospective study of the Sofia aspiration catheter (15). Another study showed an mFPE rate of 67% when using the Sofia Plus catheter with a soft-tipped, braided distal lumen of 0.070-inch (15). The retrospective study by Alawieh et al. showed that the rate of reperfusion at first pass ranged from 55 to 68% among different types of aspiration devices (5 MAX 0.054” 55%, ACE 0.060” 63%, ACE 64.064” 63%, and ACE 68.068” 68%) (7). The rate of mFPE was 64.6% in our study, which was similar to the aforementioned. In addition, the rate of achieving complete reperfusion was 43.5% in our study and was comparable to the 47% reported in a previous study using Sofia Plus (8). Even when compared with recent ASTER-2 trial, the rate of FPE and mFPE in our study was higher than 40.9 and 53.7% rates reported for the combined approach group, and 33.7 and 44.6% in the SR group (12). The favorable outcome rate was 62.3% in this study, and it was acceptable compared with other studies (1). The overall rate of procedure-related complications including arterial perforation, dissection, and distal embolization was 10% in this study, which was comparable to 7.1% in a meta-analysis of Sofia use (11), and lower than 13% in the aspiration arm of the COMPASS trial (6), 25.1% in the combined approach and 24.3% in SR arm in ASTER-2 (12). Also, the rate of mortality (13.8 vs. 19%) and sICH (3.8 vs. 5.8%) was similar to the recent meta-analysis of Sofia use (11). Thus, despite a relatively larger bore, the Wallaby aspiration catheter system did not increase safety events.

## Limitations

The limitations of this study include the relatively small sample size, and the study was single-armed without direct comparison with other aspiration devices. Also, the study may be subject to selection bias as aspiration catheter selection in each case was discretionary. SNAKE technique is one of the main advantages of ADAPT but was not specifically analyzed during this trial. Other detailed information of the techniques used during conversion to stent-retriever or occlusion location after the initial ADAPT were not documented but may provide more valuable information of knowing this new thrombectomy device capacity. Furthermore, patients with anterior and posterior occlusions were both recruited, potentially leading to heterogeneity of the study population.

## Conclusions

In current practice, the first-line aspiration approach with the Esperance^®^ Distal Access Catheter offers safe and efficient outcomes. The device could achieve high reperfusion rate with low requirement for rescue therapy with stent retriever.

## Data Availability Statement

The original contributions presented in the study are included in the article/Supplementary Material, further inquiries can be directed to the corresponding authors.

## Ethics Statement

The studies involving human participants were reviewed and approved by Xuanwu Hospital of Capital Medical University, Anhui Provincial Hospital of Anhui Medical University, Sichuan Provincial People's Hospital, The First Affiliated Hospital of Wannan Medical College, Cangzhou Central Hospital, Xinqiao Hospital, The Second Affiliated Hospital, Eastern Theater General Hospital of the Chinese People's Liberation Army, Drum Tower Hospital of Medical School of Nanjing University and General Hospital of Northern Theater Command. The patients/participants provided their written informed consent to participate in this study.

## Author Contributions

All authors listed have made a substantial, direct, and intellectual contribution to the work and approved it for publication.

## Funding

This study was funded by Shanghai Wallaby Medical Technologies Co., Inc.

## Conflict of Interest

The authors declare that the research was conducted in the absence of any commercial or financial relationships that could be construed as a potential conflict of interest.

## Publisher's Note

All claims expressed in this article are solely those of the authors and do not necessarily represent those of their affiliated organizations, or those of the publisher, the editors and the reviewers. Any product that may be evaluated in this article, or claim that may be made by its manufacturer, is not guaranteed or endorsed by the publisher.
