# Supplementary material for: A Prospective, Multicenter, Single-Group Target-Value Clinical Trial to Evaluate the Safety and Efficacy of a Large Bore Aspiration Catheter System for the Endovascular Treatment of Acute Ischemic Stroke
Source: Front Neurol. 2022 Jun 9;13:864563. doi: 10.3389/fneur.2022.864563 (PMC9218266; doi:10.3389/fneur.2022.864563)
Supplement: Supplementary file 1 [file Data_Sheet_1.docx]

**Inclusion Criteria**

1. Age ≥ 18

2. Subjects with acute ischemic onset stroke and preoperative imaging (CTA/DSA/MRA) demonstrating anterior circulation occlusion (internal carotid artery, middle cerebral artery M1segment or M2 segment, M2 segment according to physician's judgement) or posterior circulation vascular occlusion

3. NIHSS score ≥6 at admission and mRS≤2 before stroke onset

4. Within 24 hours from stroke onset. Head CT or MR examination should be performed, and ASPECTS (of patients with acute anterior circulation stroke) or pc-ASPECTS (assessed in patients with acute posterior circulation stroke) need to be ≥ 6 points; if the ASPECTS meet the conditions after head CT or MR examination, CTP or MRP examination is required to assist in the evaluation of the infarct core and penumbra volume if the time from the onset to the groin puncture is ≥ 6 hours, ≤24 hours for anterior circulation stroke

5. Informed consent form signed by subjects or their legal guardian

**Exclusion Criteria**

1. Imaging demonstrating anterior circulation large area cerebral infarction (imaging examination showed infarct volume ≥ 70ml or infarct area> 1/3 MCA blood supply area) or focal space occupying lesion effect significantly led to the displacement of midline structures

2. Intracranial hemorrhage or subarachnoid hemorrhage confirmed by imaging examination (Excluding microhemorrhage on SWI or assessed by clinical investigator)

3. Imaging showing simultaneous acute occlusion of bilateral carotid arteries; tandem lesions with simultaneous occlusion of intracranial and extracranial vessels

4.Subjects with disturbance of consciousness due to posterior circulation vascular occlusion, NIHSS consciousness level score (1a=3)

5. Angiography shows occlusion of an intracranial artery due to arterial dissection or arteritis

6. The study device cannot reach the target vessels due to tortuous vascular approach or other difficulty

7. Subjects known allergic or resistant or contraindicate to one or more of the following: antiplatelet agents/anticoagulation/contrast agents and/or anesthetics

8. Subjects known to be allergic to study medical devices and related products; (allergic to materials such as nickel-titanium or its alloys)

9. Subjects known active bleeding or known bleeding tendency (eg, preoperative anticoagulant therapy or coagulation dysfunction before operation, INR > 3.0, except for subjects receiving thrombolysis before surgery)

10. Subjects with the history of platelet count lower than 40×10^^9^/L

11. Subjects with history of severe heart failure, severe liver failure or severe kidney failure.

12. Subjects with ST-segment elevation myocardial infarction or severe infection (endocarditis or sepsis)

13. Subjects with major surgery within the past 1 month

14. Subjects with gastrointestinal or urinary bleeding within 1 month

15. Subjects with hypertension that cannot be of controlled with drug treatment (systolic blood pressure persists ≥185 mmHg or diastolic blood pressure persists ≥110mmHg)

16. Subjects with blood glucose concentration < 2.7 or > 22.2 mmol/L after treatment

17. Life expectancy <3 months

18. Female subjects of childbearing age should not be pregnant or plan to be pregnant for duration of clinical study (by asking about medical history)

19. Subjects with dementia or mental illness who cannot complete the assessment of neurological function

20. Subjects who are participating in clinical studies of other drugs or devices

21. Subjects not included for other reasons from the investigators
